# Genomic view on the peopling of India

**DOI:** 10.1186/2041-2223-3-20

**Published:** 2012-10-01

**Authors:** Rakesh Tamang, Kumarasamy Thangaraj

**Affiliations:** 1 CSIR-Centre for Cellular and Molecular Biology, Uppal Road, Hyderabad, 500 007, India

**Keywords:** Admixture, caste, Indians, mtDNA, tribe, Y-chromosome

## Abstract

India is known for its vast human diversity, consisting of more than four and a half thousand anthropologically well-defined populations. Each population differs in terms of language, culture, physical features and, most importantly, genetic architecture. The size of populations varies from a few hundred to millions. Based on the social structure, Indians are classified into various caste, tribe and religious groups. These social classifications are very rigid and have remained undisturbed by emerging urbanisation and cultural changes. The variable social customs, strict endogamy marriage practices, long-term isolation and evolutionary forces have added immensely to the diversification of the Indian populations. These factors have also led to these populations acquiring a set of Indian-specific genetic variations responsible for various diseases in India. Interestingly, most of these variations are absent outside the Indian subcontinent. Thus, this review is focused on the peopling of India, the caste system, marriage practice and the resulting health and forensic implications.

## Review

### Introduction

India is well-known for its human and geographical diversities. It has a variety of landscapes ranging from desert to evergreen forest, fertile plains to southern dry plateaus, shallow swamps to deep bays, and lowlands to the high Himalayas. They all give refuge to diverse populations of humans, plants, animals and microbes. India also has extensive river systems that feed the fertile plains and provide adequate food. It is bordered by Nepal, China and Bhutan in the north, Burma and Bangladesh in the east, and Pakistan in the west. There are 4,635 anthropologically well-defined human populations; 532 are tribes, including 72 ancestral tribes with the hunter and gatherer lifestyle [1]. The size of these populations ranges from a few hundred to several millions. The total population count of India has reached 1.21 billion [2].

**Figure 1:**
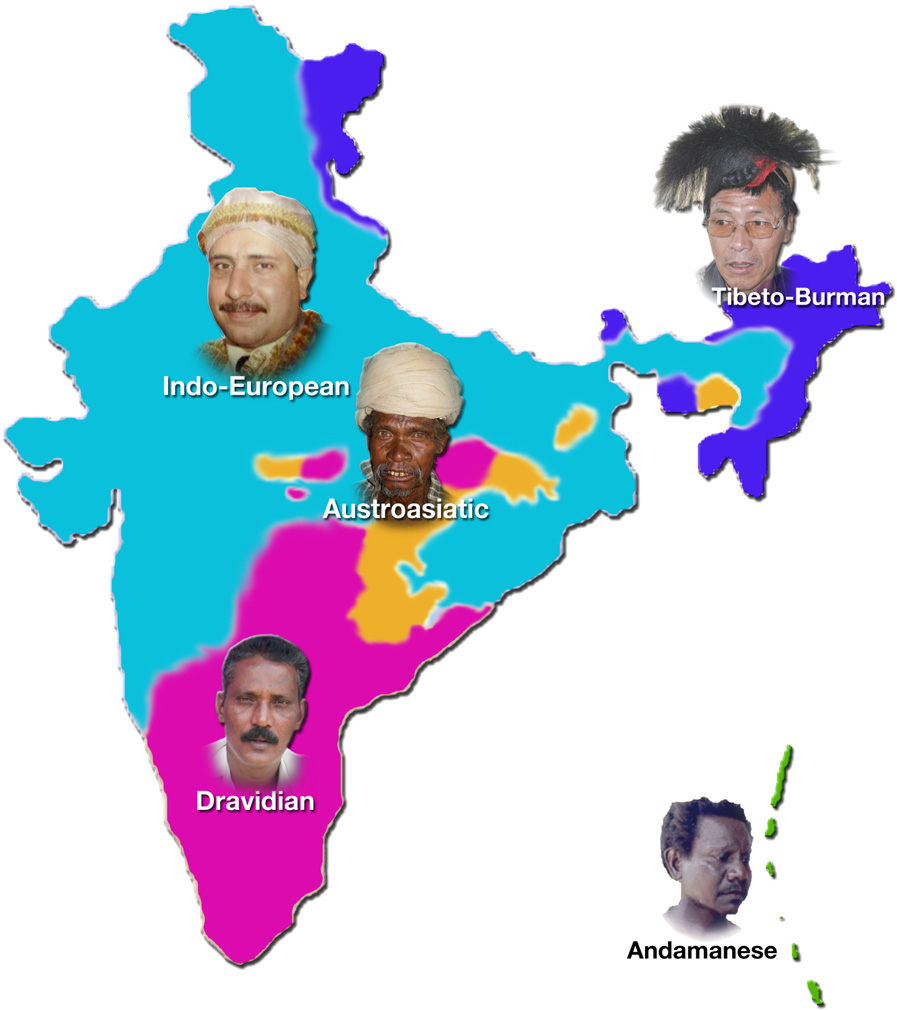
Geographical distribution of different language families in India.

Linguistically, Indians are classified into four major language families; Indo-European, Dravidian, Austroasiatic and Tibeto-Burman (Figure 1). Indo-European is the most widely spoken language family in India, particularly in northern, central and western India. Dravidian speakers are mainly confined to southern parts of the country. Austroasiatic speakers are dispersed mostly in the central and eastern parts, while the Tibeto-Burman speakers are concentrated in and around the foothills of the Himalayas and north east states (Figure 1). In addition to these major language families, there are a few isolated languages, such as Andamanese [3,4], spoken by the enigmatic tribal populations of Andaman and Nicobar Islands, and Nihali spoken in the pocket of the Western Ghats.

There are various evidences supporting the peopling of India by the early modern humans. It is well established that the modern human originated in Africa about 200,000 years before present (YBP) [5,6]. They started migrating out-of-Africa between 55,000 and 85,000 YBP. There are several thoughts regarding the cause and timing of this migration. One compelling view being put forward is based on geological finding. It states that there was a mega drought in East Africa between 135,000 and 75,000 YBP, when the water volume of Lake Malawi was reduced by at least 95% [7]. The timing of this mega drought corresponds with the timing of the exodus of anatomically modern humans out-of-Africa along the southern coastal route [4,8]. The firm establishment of the southern coastal route of modern human migration reveals India as a major corridor for early human migration. The anthropological, historical, linguistic and genetic evidence for early peopling of India is found imprinted all over the country.

Recently, archaeological evidence supporting the early peopling of India was discovered in the Kurnool district of Andhra Pradesh, one of the southern Indian states [9,10]. This study shows that the country was inhabited by modern humans before and after the Toba eruption around 74,000 YBP. The evidence is in the form of stone tools. The stone tools of this study most likely resemble contemporaneous *Homo sapiens* technologies in Africa. Further, a partial cranium recovered from Narmada Basin was dated back to around 300,000 to 250,000 YBP [11,12]. Over the past two decades, several independent studies have been carried out in various Indian populations with ancient and modern DNA using haploid and diploid markers. Almost all the studies found signs of early settlement by the first group of modern human venturing out-of-Africa and very recent gene flow from west and east Eurasia [4,8,13-34].

There has been tremendous interest among historians, archaeologists, anthropologists, linguists and geneticists to understand the unique structure of Indian populations and their affinities with the rest of the world. Most importantly, researchers working on various diseases often find that disease-causing genetic variations are different in Indian populations. During the last two decades, many exciting observations have been made regarding Indian people by several investigators; however, these findings have remained scattered. Thus, we have made an attempt to present an overview of the peopling of India, the caste system, endogamous marriage practice, and the resulting health and forensic implications.

### Evidence of the first out-of-Africa human in the Indian subcontinent

An interesting finding about the Indian population is the evidence of the early settlement in Andaman and Nicobar Islands by a group of modern humans, whose ancestors made the first journey out-of-Africa. The Andaman and Nicobar Islands are located in the Bay of Bengal (Figure 1). The study of the isolated people of these islands provides important clues to the evolution and dispersal of early modern humans. There live two groups of tribes in the Andaman and Nicobar Islands: those who share physical features with African pygmies and other similarly featured Asian people, such as short stature, dark skin, peppercorn hair, and scant body hair; and those who share physical features with the Chinese, Malays and Burmese. Linguistically, the people of Andaman Islands can be divided into Little Andaman and the Great Andaman groups. The Little Andaman group is divided into three branches, the Onge, Sentinelese and Jarawa. The Great Andaman group is divided into the Northern and Southern groups [3].

There are fewer archaeological and genetic records on the origin of Andaman Islanders. Our group [35] and Endicott and his group [15] undertook parallel studies on the people of Andaman and Nicobar Islands. The latter group dealt with the ancient remains of these populations using mitochondrial DNA (mtDNA), while we studied modern DNA using mtDNA and Y-chromosomal markers [4,35]. Surprisingly, both of the studies established Andamanese affinity with Asian populations. Subsequently, we performed a high resolution study using complete mtDNA of the Onge and Greater Andamanese. We found unique mutations in their mtDNA. Hence, we have assigned two novel haplogroups, M31 and M32, and estimated the age of these haplogroups to be about 65,000 years. This suggests an ancient origin of the people of Andaman and Nicobar Islands and long-term isolation [4]. Even though researchers have come forward with different time estimates on the peopling of Andaman and Nicobar Islands [36], the early peopling of this region cannot be ruled out [36,37]. Recently, our study using 560,123 autosomal SNPs suggested a unique genetic identity of the Andamanese and ancient isolation from mainland south Asian populations. However, they shared genetic affinities with ancient south Indians (ASI) [30].

### The caste system in India

India is a land of social stratifications, such as castes, tribes and religious groups. Although the precise date of origin for the caste system in India is unclear, the written evidence about the organisation of the caste system exists in the *Rig Veda*, which was written between 1700 and 1100 BC [38]. Caste is a social hierarchy based on occupation. There are four broad categories of castes in Hindu society: Brahmin, Kshatriya, Vaishya and Sudra. Each caste is known to perform a specific duty. Brahmins perform rituals and are in charge of teaching society; Kshatriyas are rulers and warriors, and are involved in ruling and defending the territories; Vaishyas are cultivators and businessmen; Sudras rank last in society and are labourers by profession. Each caste is further subdivided into smaller units generally known as subcastes, which in turn are further divided into multiple exogamous clans known as Gotras. The caste system governs all social, religious and economic activities of the people. The long-term social boundaries and endogamy practice among all social groups has given birth to diverse, population-specific social traditions and the development of distinct linguistic dialects [39]. The divergent endogamous cultural and social structures are helpful in understanding genetic variation among the populations and their ancestry [39].

There are several genetic studies on the caste systems in India based on mtDNA and Y-chromosome markers [16,19,33,34,40-44]. These studies reveal that the origin of the caste system is mainly rooted in male-mediated Indo-Aryan migration that pushed indigenous Dravidian speaking populations towards southern India and Sri Lanka, and suggest that the Indo-Aryans established themselves as the upper caste [39]. Further, it has been shown that the caste populations are closer to Europeans and Central Asians and differ significantly from tribal populations [34,39,43,45]. These studies succeeded in drawing a trend to investigate the integrity of the caste system. Most of the researchers started by including the Indo-Aryan invasion concept in their studies, assuming that it was a universally accepted and proven fact [33,46-50].

#### Emergence of caste system in India and its amalgamation with the waves of migrations

**Figure 2:**
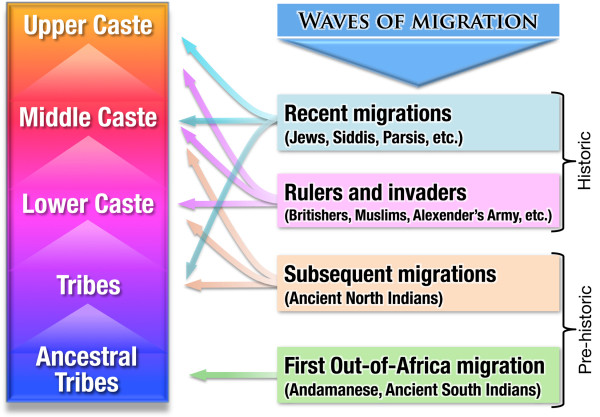
Schematic showing the emergence of the caste system in India and its amalgamation with the waves of migrations.

A noteworthy view can be put forward on the development and maintenance of the caste system on the basis of genetic observations (Figure 2) [33,51,52]. The ancestral tribes might have given birth to various subtribes over time. Some of the subtribes might have migrated and gradually established themselves as lower caste groups through better knowledge of procuring necessary resources. Further, the empowerments of some of the lower caste group might have helped them to establish themselves as middle caste groups. Increased mastery over technological and economic measures among some of the middle caste group might have facilitated attainment of the upper caste level (Figure 2), thus giving rise to a complete caste system. Hence, a person’s profession became the symbol of the caste to which he or she belonged. As time passed, the caste system might have fortified itself in society by dividing populations into several endogamous pockets, and has undoubtedly played an essential role in shaping present day Indian genetic architecture. Furthermore, India has witnessed several waves of immigrations. The immigrants were absorbed into different hierarchal levels of the caste system (Figure 2).

#### No support for the Aryan invasion

Even though there is a continued debate on the Aryan migration into India, detectable gene flow from west Eurasia has been shown by many studies [13,16,23,24,30-32,44,51,53]. Interestingly, we have detected gene flow from the west prior to the Aryan invasion [30,32]. There is now universal agreement that various Indian populations share a common late Pleistocene maternal and paternal ancestry, along with detectable east and west Eurasian ancestries [31,54]. Using hundreds of thousands of autosomal markers, we illustrated that the Indian populations have two major distinct ancestry components; one restricted to southern India, the second one restricted to the northern region of India [30,32]. It is noteworthy that both of the ancestry components show higher haplotypic diversity than those predominant in west Eurasia [32]. This rejects the idea of an Aryan invasion/migration and suggests an ancient demographic history and/or higher long-term larger effective population size in India than in west Eurasia.

### Genetic structure of ancient India

#### Indigenous population

During the past two decades, we have witnessed remarkable advancements in technology. We have advanced from low resolution genetic markers to high throughput whole genome sequencing. Despite these advancements, studies using high density markers were lacking in the Indian scenario. Therefore, we made an attempt to extensively study various Indian populations using Affymetrix (SNP 6.0) array [30].

We predicted that the present day Indian populations might have arisen from a relatively small group of ancestors with limited gene flow and long term isolation. We also predicted that there might have been two ancestral groups in prehistoric India: an ancestral North Indian (ANI) population distantly related to those in the Middle East, Central Asia and Europe (30% to 70%), and an ASI population not related to groups outside India. We estimated that the ASI populations showed 39% to 71% ANI ancestry. Thus, the extant Indian populations are admixtures of both ANI and ASI. Interestingly, indigenous Andaman Islanders are the only ASI-related groups without ANI ancestry [30].

#### Recent migrants

In addition to the large number of indigenous populations, India has experienced immigration of several populations in the past, further adding to the complexities of Indian population structure. Among the recent migrants, we have extensively studied Siddi, Muslim and Jewish populations inhabiting India.

The Siddis are mainly found in three Indian states - Gujarat, Karnataka and Andhra Pradesh -and have typical African features such as dark skin, curly hair, broad nose, and so on. Historically, it is known that they were brought to India by Portuguese traders between the 17^th^ and 19^th^ centuries and sold to the Nawabs and the Sultans of India to serve as soldiers and slaves. Earlier, we showed the presence of a Y-chromosome *Alu* insertion; an African-specific marker, in 40% of Siddis [55]. The mitochondrial DNA hypervariable sequence also confirmed the Siddis’ affinity with Africans. However, there was no high resolution study pertaining to the origin, affinity and genetic structure of the Indian Siddis [56-58]. In an attempt to reveal these issues together, we screened the Siddi populations from the Junagarh district of Gujarat and Uttara Kannad district of Karnataka using mtDNA and Y-chromosomal and high density autosomal markers. Along with Siddis, six populations with geographical proximity to Siddis have also been studied to capture a scenario of gene flow. Our analysis revealed that the Siddi population has a combined ancestry (that is, 70% African and 30% Indian and European) [24]. We further estimated that the Siddis have admixed with the neighbouring Indian populations for about 200 years ago (eight generations). Our genetic finding coincides with the historical record of the arrival of Siddi people in India [24].

Y-chromosome results revealed the presence of African-specific haplogroups. Further, extensive investigation on Y-STRs revealed that the Siddis are the direct descendants of the Bantu-speakers of sub-Saharan Africa. We observed an Indian-specific gene pool in Siddis but the Siddi-specific gene pool was not observed in neighbouring Indian populations. This firmly suggests the unidirectional gene flow from the Indian population to the Siddis, confirming the rigidity of the Indian social structure [24]. We also predicted that the approximate number of males Siddis who reached India between the 17^th^ and 19^th^ centuries was about 1,500.

Another well-documented recent migration is that of the Muslim population. The Arab Muslims established their first kingdom in India by conquering Sindh in 711 AD [59,60]. During the 13^th^ century, a Turkic kingdom was established in Delhi and in the 16^th^ century the famous Mughal Empire appeared in India. The Muslim immigrations were especially male-mediated in the form of invaders from Iran and Arabia. The majority of the present day Indian Muslims are believed to be the descendants of converts from local Hindu (caste and tribal) populations. Thus, we undertook an extensive study among Indian Muslims from diverse geographical regions to trace their genetic structure, origin and affinity using Y-chromosome and mtDNA markers. Even though we observed their genetic affinity with indigenous Indian non-Muslim populations, a small frequency of the Middle Eastern ancestry was also noticed. Therefore, the genetic analysis of Indian Muslims shows the spread of Muslims in India was mainly mediated by cultural adaptation linked with minor gene flow from the Middle East [61,62].

### Discrepancy between linguistic affiliation and genetic signature

Each linguistic group in India has a strong genetic affinity between its members. Therefore, any recent change in language could be reflected in the genome. When people move from one place to another, as per human tendency, they try to adapt themselves to the new place. Over time, some of the migrants adopt the local language for better communication and, in turn, for better living. Thus, language shift is a phenomenon where a new language is adopted by a population with virtually no influence on their genetic make-up. There are several examples of language shift around the world [63-65]. In India, we have documented two case studies, where linguistic affiliation and gene pool reveal different scenarios.

In India, the first evidence of inconsistency between language and gene pool was noticed by us among the Hindi (Indo-European)-speaking Mushar population [52]. mtDNA and Y-chromosomal datasets of this population were compared with those of the neighbouring Indo-European- and Austroasiatic-speaking populations of similar traditional social status. mtDNA analysis revealed the presence of both Austroasiatic and Indo-European haplogroups. However, the Austroasiatic-specific haplogroups were observed in higher frequencies. A similar scenario was noticed with Y-chromosomal marker data. Further, principal component analysis revealed a closer genetic affinity to the Austroasiatic (Mundari) populations than to the neighbouring Hindi-speaking populations. This showed that the Mushar population was originally an Austroasiatic group (genetically) but linguistically, it is an Indo-European population [52]. The rapid loss of original tongue was facilitated by the schooling of Mushar children in the Indo-European language-based schools [52].

The second evidence of discrepancy was observed among the Bharia tribe of the central Indian state of Madhya Pradesh. We carried out an extensive characterization of genetic ancestry of Bharia, a Dravidian speaking group, and its Indo-European-speaking neighbours (Bhil and Sahariya) along with other Austroasiatic-speaking groups. mtDNA analysis showed the presence of lineages that are commonly found among the Austroasiatic groups [54,66]. In addition, Y-chromosomal biallelic markers also revealed a high frequency of the Austroasiatic-specific haplogroup in Bharia. Further, principal component analysis showed a strong genetic affinity of Bharia with the Austroasiatic (Munda) group [23]. The gene flow from the Munda group was also confirmed by analysis of the Y-STRs haplotype sharing. The autosomal markers also established the Austroasiatic gene pool among Bharia. Hence, in a landscape like India, the linguistic label of a population does not unequivocally follow the genetic footprints [23].

#### The origin of Austroasiatic speakers

There are 104 million Austroasiatic speakers around the globe. There exist two major branches of the Austroasiatic language in India. The Munda branch is mostly spoken by eastern, northeastern, and central Indians, and the Khasi-Aslian branch is located in Meghalaya and (Andaman) Nicobar Islands [67]. There are disputes on the geographic origin of the Austroasiatic language family [27,68-71]. The first hypothesis places the origin of Austroasiatic speakers in Southeast Asia with a later dispersal to South Asia during the Neolithic period [72]. The second hypothesis advocates pre-Neolithic origins and dispersal of this language family from South Asia [68]. The main lacuna in previous studies was the absence of extensive sampling from both Southeast Asia and India, and a smaller number of markers used. Therefore, to test the two alternative models, we performed combined analysis of uniparentally inherited markers and one million common SNP loci from the nuclear genome [27]. Our result showed higher diversity of haplogroup M95-O2a with an older coalescent time of 17,000 to 28,000 years ago in Southeast Asia samples than in their Indian counterparts, supporting the first of the two hypotheses. A principal component analysis and structure-like analysis on autosomal loci showed a more complex population history of Austroasiatic speakers in India. Thus, we propose that the Austroasiatic speakers in India dispersed from Southeast Asia, followed by extensive sex-specific admixture with local Indian populations [27].

### Endogamy as a barrier to the general perception about patrilocality and matrilocality

The presence of both patrilocal and matrilocal societies further adds to the complexities of the Indian social structure. The societies in which a woman moves to her husband’s home after marriage are called patrilocal societies, and the societies in which a man moves to his wife’s home is a matrilocal society. Thus, these social customs are expected to have an impact on the patterns of genetic variation among the population of a given region. It is indeed observed that there is localization of male-specific Y-chromosomal variants and wide dispersal of female-specific mtDNA variants in patrilocal societies and *vice versa* in matrilocal societies [73-75]. However, Indian societies are strictly endogamous and have very rigid social customs. Therefore, marriage always takes places within a population, and these different patterns of genetic variations among patrilocal and matrilocal Indian populations are not the expected Indian scenario. We, along with other researchers [76], have analysed 10 patrilocal and five matrilocal Indian populations. We collectively suggest that there is an insignificant difference between the patrilocal and matrilocal societies. Hence, the patterns of genetic variation in humans are not always universal, but depend on local cultural practice [76].

### Unique genetic variations in Indian populations and their implications

#### Implications in health and disease

Several boundaries acting at different levels within the Indian populations have added to the human diversity in the country. Indian populations have been shaped by a long-term genetic isolation of different populations that predates the caste system in India. The allele frequency differences between the groups in India are larger than those in Europe, reflecting strong founder effects whose signatures have been preserved for thousands of years due to firm social customs and strict endogamy. We therefore predict that there is an excess of population and region-specific diseases in India. This prediction is well correlated with several population- or region-specific diseases and medical conditions, for example, Madras motor neuron disease [77,78], Handigodu disease [79,80], and pseudocholinesterase deficiency among Vysyas [81,82]. Further, our study on high density markers in 142 samples from 30 ethnic groups in India predicted the positive selection of *MSTN* and *DOK5* genes, which have potential implications in lipid metabolism and the aetiology of type 2 diabetes in Indians [32].

Our previous study revealed that the deletion of 25 bp in the myosin binding protein-C3 gene (*MYBPC3*) is associated with inheritable cardiomyopathies in India due to skipping of wild type exon 33 [83]. We also demonstrated that this mutation accounts for 45% of cardiac deaths in India from sudden heart attack. This mutation is widely distributed (4.5%) across India, but was not found in the tribes of Andaman and Nicobar Islands and northeastern populations of India, who have different ancestry. We have screened this mutation in the worldwide population, representing 26 countries, and found this mutation only in the populations of India, Pakistan, Sri Lanka, Indonesia and Malaysia (South and Southeast Asian countries). We further estimated that this mutation probably originated in India about 33,000 years ago.

We have also observed the implications of the admixture of ethnically diverse populations, who have undergone selection processes through environmental influence. The best example is the Siddis, the Indo-African population, whose admixture with the neighbouring Indian populations has remarkable medical and social implications. The A-variant of the *G6PD* gene that gives protection against malaria is commonly found in Africa. Even though the Siddis have African origin, the A-variant was found only in 10% of the Siddi individuals. This is primarily because of their admixture with the neighbouring Indian populations, which in turn has diluted the frequency of the A-variant. This makes the present day Siddis more susceptible to malaria than their African ancestors.

The differences in disease rates between populations are largely due to the frequency differences in disease-causing genetic variants. It is said that these genetic variants occur frequently on chromosome segments inherited from the ancestral population with the higher disease-variant frequency [84]. Thus, efficient mapping of disease-causing variants are improved if the divergence between the ancestral populations is large. A large difference in the prevalence of the disease between the ancestral and derived populations adds to the successfully mapping of disease-causing variants, which is more often observed in the case of the admixed populations [85]. A study on the Indian populations spanning African descendants has shown the importance of admixture in mapping the diseases [85]. A significant enrichment of processes related to ion-channel activity and cadherin genes was found. Selection in ion channel genes among populations of African ancestry can be explained by the fact that this population resides in an extremely saline region of the country [85]. This finding was similar to results regarding African Americans, where the family of genes related to ion channels, cadherins, and calmodulins, had disproportionate influence on hypertension and associated complications [86].

#### Forensic implication

The structure of the Indian population also has implications in forensic genetics. Highly polymorphic STR markers provided by Applied Biosystems, Foster City, CA, USA, and Promega, Madison, WI, USA are being extensively used for medicolegal purposes. The abilities of these markers to differentiate individuals are quite efficient. However, the strict endogamy practices and small population size of some Indian populations has presented a potential challenge to the efficiency of these methods. For example, three Y-STRs loci were found to be monomorphic for Onges, and one for Nicobarese people [35]. There may be more cases in different endogamous Indian populations. Therefore, it is difficult to distinguish individuals using only commercially available marker sets. Thus, further refinement of commonly used STRs is necessary to differentiate each individual to its best possible resolution.

The amelogenin gene, which is present on both X and Y chromosomes differs in size. This fact has been widely used in forensic casework and prenatal diagnosis to identify sex; however, regarding the same marker among the diverse Indian populations, we have observed a deletion of Y-chromosome-specific amelogenin in 1.85% of Indian males [87]. These individuals would have been considered female through routine analysis, thus creating a massive misinterpretation of fact. Therefore, considering the false consequences of the result obtained by only using the amelogenin marker, we suggest the use of additional Y-chromosome markers for unambiguous gender identification.

In a mass disaster or crime investigation, genetic markers play a major role in victim identification. We have shown earlier that every Indian population forms a separate cluster [30]. Therefore, by using a larger number of SNPs (for example, Affymetrix 6.0 array), it is possible to distinguish every population involved. The Y-chromosomal and mitochondrial markers used in classifying the individuals into haplogroups can also be used to elucidate additional facts.

## Conclusions

Based on the extensive genetic studies carried out on different Indian populations, we infer that each of them is a genetically distinct ethnic population in part due to high levels of endogamy. High resolution genetic studies revealed the *in situ* origin of several deep-rooting mtDNA lineages in India, suggesting that Indian populations are genetically unique and harbour the second highest genetic diversity after Africans. The genomic complexity brought on by endogamous practice for thousands of years, language shifts and sex-specific admixture are highly challenging to study and need further, extensive genetic characterization. The complex genomic architecture of Indian populations adds difficulty in understanding diseases and implementing preventive measures. The accumulation of various mutations due to endogamy leads to recessive diseases in the Indian population, which further increases the total disease burden of the country. Several questions pertaining to the origin of the caste system, the arrival and spread of the major language families and finding disease-associated genetic variants require effective approaches combining various disciplines such as archaeology, historical linguistics, genetics, bioinformatics and pharmacology. Even though studies with high density markers have added much to the knowledge about the Indian populations, whole genome approaches are expected to answer many of the existing questions.

## Abbreviations

ANI: ancestral North Indian; ASI: ancestral South Indian; bp: base pairs; mtDNA: mitochondrial DNA; SNP: single nucleotide polymorphism; STR: short tandem repeat; YBP: years before present.

## Competing interests

The authors declare that they have no competing interests.

## Authors’ contributions

RT and KT wrote the manuscript and performed graphical work. Both the authors read and approved the final manuscript.
